# Wildfire, Smoke Exposure, Human Health, and Environmental Justice Need to be Integrated into Forest Restoration and Management

**DOI:** 10.1007/s40572-022-00355-7

**Published:** 2022-05-07

**Authors:** Savannah M. D’Evelyn, Jihoon Jung, Ernesto Alvarado, Jill Baumgartner, Pete Caligiuri, R. Keala Hagmann, Sarah B. Henderson, Paul F. Hessburg, Sean Hopkins, Edward J. Kasner, Meg A. Krawchuk, Jennifer E. Krenz, Jamie M. Lydersen, Miriam E. Marlier, Yuta J. Masuda, Kerry Metlen, Gillian Mittelstaedt, Susan J. Prichard, Claire L. Schollaert, Edward B. Smith, Jens T. Stevens, Christopher W. Tessum, Carolyn Reeb-Whitaker, Joseph L. Wilkins, Nicholas H. Wolff, Leah M. Wood, Ryan D. Haugo, June T. Spector

**Affiliations:** 1 Dept. of Environmental & Occupational Health Sciences, University of Washington, 3980 15th Ave NE, Seattle, WA 98105 USA; 2 School of Environmental and Forest Sciences, University of Washington, Seattle, USA; 3 Dept of Epidemiology, Biostatistics & Occupational Health, McGill University, Montreal, Canada; 4 The Nature Conservancy, Arlington, USA; 5 Applegate Forestry, LLC, Corvallis, USA; 6 British Columbia Centre for Disease Control, Vancouver, Canada; 7 USDA Forest Service, Pacific Northwest Research Station, Wenatchee, WA USA; 8 Washington State Department of Ecology, Lacey, USA; 9 Dept. of Forest Ecosystems and Society, Oregon State University, Corvallis, USA; 10 California Department of Forestry and Fire Protection, Sacramento, USA; 11 Department of Environmental Health Sciences, Fielding School of Public Health, University of California Los Angeles, Los Angeles, USA; 12 Partnership for Air Matters, Tribal Healthy Homes Network, Seattle, USA; 13 Department of Biology, University of New Mexico, Albuquerque, NM USA; 14 Dept. of Civil & Environmental Engineering, University of Illinois at Urbana-Champaign, Champaign, USA; 15 Safety & Health Assessment & Research for Prevention Program, Washington State Department of Labor and Industries, Tumwater, USA; 16 Interdisciplinary Studies Department, Howard University, Washington, DC USA; 17 Evan’s School of Public Policy and Governance and The Department of Global Health, University of Washington, 3980 15th Ave NE, Seattle, WA 98105 USA

**Keywords:** Wildland fire, Public health, Air quality, Smoke, Exposure, Ecological restoration, Prescribed burning, Environmental justice, Interdisciplinary, Collaborative partnerships

## Abstract

**Purpose of Review:**

Increasing wildfire size and severity across the western United States has created an environmental and social crisis that must be approached from a transdisciplinary perspective. Climate change and more than a century of fire exclusion and wildfire suppression have led to contemporary wildfires with more severe environmental impacts and human smoke exposure. Wildfires increase smoke exposure for broad swaths of the US population, though outdoor workers and socially disadvantaged groups with limited adaptive capacity can be disproportionally exposed. Exposure to wildfire smoke is associated with a range of health impacts in children and adults, including exacerbation of existing respiratory diseases such as asthma and chronic obstructive pulmonary disease, worse birth outcomes, and cardiovascular events. Seasonally dry forests in Washington, Oregon, and California can benefit from ecological restoration as a way to adapt forests to climate change and reduce smoke impacts on affected communities.

**Recent Findings:**

Each wildfire season, large smoke events, and their adverse impacts on human health receive considerable attention from both the public and policymakers. The severity of recent wildfire seasons has state and federal governments outlining budgets and prioritizing policies to combat the worsening crisis. This surging attention provides an opportunity to outline the actions needed now to advance research and practice on conservation, economic, environmental justice, and public health interests, as well as the trade-offs that must be considered.

**Summary:**

Scientists, planners, foresters and fire managers, fire safety, air quality, and public health practitioners must collaboratively work together. This article is the result of a series of transdisciplinary conversations to find common ground and subsequently provide a holistic view of how forest and fire management intersect with human health through the impacts of smoke and articulate the need for an integrated approach to both planning and practice.

**Supplementary Information:**

The online version contains supplementary material available at 10.1007/s40572-022-00355-7.

## Introduction

Fire is a globally significant phenomenon affecting both human and wildland ecosystems [1]. Area burned in the western United States (US) has increased steadily in recent decades despite suppression efforts [2, 3]. As contemporary wildfires grow larger and more severe, there is mounting recognition of an urgent need to integrate human health and ecosystem management perspectives due to the well-recognized and pervasive effects of smoke on human populations. Seasonally dry forests of the western US are iconic examples of this modern issue, where several key factors align: increasing area burned and increasing area burned at high severity [3, 4], anthropogenic climate change [5], and widespread calls to restore forests and protect ecosystem services, native biodiversity, and human communities [6].

We define wildland fire as any non-structural fire that occurs away from developed areas, including fires that are intentionally burned under prescription (prescribed fire) and unplanned fire events (wildfire). Some wildfires occur under conditions conducive to achieving ecological objectives if monitored and allowed to continue burning rather than being suppressed (managed wildfire). Prescribed fires and managed wildfires are conducted under targeted weather and fuel conditions to achieve land management objectives and limit smoke exposure as much as possible [7]. However, smoke exposure from any fire event can adversely affect exposed populations [8, 9]. The health impacts of smoke exposure include, but are not limited to, exacerbation of respiratory diseases such as asthma and chronic obstructive pulmonary disease (COPD), irritation of the eye, nose, and throat, and adverse birth outcomes. These impacts are discussed further in the section “*Health Effects of Wildfire Smoke Exposure*” below.

Inequalities exist in individual susceptibility and exposure to smoke, and thus the intersections between wildland fire, forest health, and public health also expose potential environmental injustices. These inequalities are elaborated upon in the section “*Affected Populations**.*” To address this complex issue and advance research and practice, scientists, managers, and policymakers must approach wildland fire, smoke exposure, human health, and environmental justice more holistically. This could include increasing research with disproportionately impacted communities to identify potential solutions and strategies for fostering resilience and ensuring that these affected communities are centered in planning and action. Academic and region-based groups and institutions have begun discussions of transdisciplinary approaches, but more rapid progress in theory, practice, and policy is needed [10–12].

The goal of this review is to articulate consensus-driven, evidence-based approaches to identify and communicate the human health and environmental justice (EJ) implications of exposure to smoke within a forest restoration and adaptive management framework. This review results from extended discussions among a transdisciplinary group of scientists and practitioners in forest ecology and management, fire science, fire safety, air quality, and public health. It provides an overview of the past, present, and future of wildland fire, forest ecology, and forest management in seasonally dry forests of the western US; summarizes existing knowledge about smoke exposures, health effects, and disproportionately affected populations; and provides transdisciplinary synthesis including consensus statements and recommendations for a path forward. 

To advance the dialogue and research agenda, we ask: (1) what are the public health outcomes of *status quo* fire and smoke management in the western US, and how might smoke emissions and associated health outcomes be reduced by alternative management scenarios? We also ask: (2) how might public health be most effectively and equitably incorporated into the management of western US forest landscapes historically maintained by fire?

For this review, we specifically focus on the potential of restorative actions in seasonally dry forests [13–15] of the western US to mitigate the increasingly negative impacts of fire on human health. Smoke crosses all boundaries, and emissions from local, regional, and international fire events all impact human health [16, 17]. However, in this manuscript, we place particular emphasis on the Pacific states of California, Oregon, and Washington, which is the geographical location referenced when “western US” is used. Our focus here provides a transdisciplinary anchor from which to establish future discussions and actions regarding trade-offs between human health, health equity, and smoke emissions from fire and forest management.

## Fire Exclusion and the Rise of Modern Western Wildfires

**Fig. 1:**
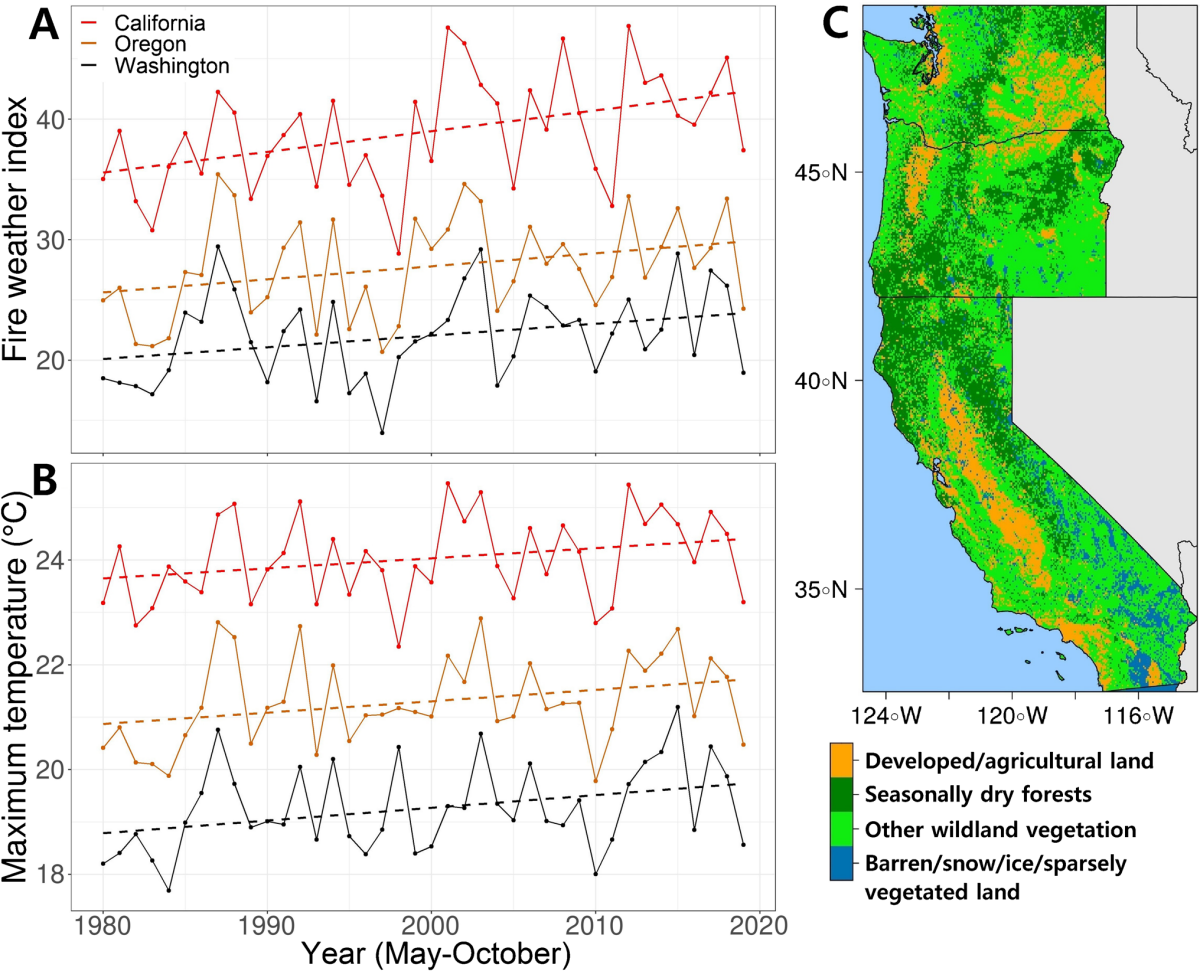
Historical weather and wildland vegetation in the western US in May-October. **A** Time series of the annual mean fire weather index and **B** the annual maximum temperatures from 1980 to 2019 within seasonally dry coniferous forests. The fire weather index is a measure of potential fire intensity based on temperature, relative humidity, wind speed, and 24-h precipitation. Only the wildfire season from May through October was plotted. **C** Fire regime map based on vegetation cover and natural fire regimes in 2019. Data downloaded from climatologylab.org/gridmet for weather data and landfire.gov/ for fire regime and vegetation cover data. Methodology described in Supplemental Text 1

Beginning in the mid-1980s, burned area in seasonally dry western US forests began a steady increase [18] despite escalating fire suppression expenditures [19]. Seasonally dry coniferous forests include pine-dominated and dry or moist mixed-conifer forests that are dry enough to burn most years during the wildfire season. Climate change contributes to increased burned area in seasonally dry forests through warmer seasonal temperatures, longer and drier summers, below-average winter precipitation, and earlier snowmelt [18, 20] (Fig. 1). Under warmer, drier, and longer fire seasons, the incidence of large wildfires in the western US has steadily increased over the last 30 years [3, 21]. Based on climate change projections, area burned in the western US is expected to double or triple by mid-century [4, 22].

Historically, fire was the dominant natural disturbance shaping western US landscapes [23, 24]. While increasing burned area is strongly associated with anthropogenic climate warming [5], humans have markedly changed native forests and associated fire regimes as characterized by fire frequency and severity. Historically, many seasonally dry western US forests experienced fire every several decades, and some burned on regular intervals of 10 years or less, with generally widespread low-severity surface fire and more localized high-severity tree-killing fire [14]. Euro-American colonization brought changes in land use and land management, including curtailed Indigenous burning that led to abrupt and persistent declines in fire frequency beginning more than 170 years ago [25]. Fire exclusion led to increased forest density and accumulation of live and dead fuels [26]. As a result, the contemporary forests and regenerating forests that are developing after recent large fires bear little resemblance to forests that developed under more characteristic fire regimes [24, 27, 28]. Fire regime characteristics include the total amount of burned area, the distribution of fire event and fire severity patch sizes, and the frequency, intensity, seasonality, and spatial distribution of fires themselves, all of which vary by forest type and physiography (Fig. 1C). Changes to characteristic fire regimes adversely impact myriad ecosystem functions that might otherwise offer social and ecological benefit [6, 14].

Recent large fire events across the western US have called attention to the need to restore frequent fire and cultural burning practices [29, 30] and to address the detrimental impacts of fire exclusion. Much can be learned from traditional Indigenous practice when it comes to burning. Native Americans in the western US traditionally used fire extensively and for a wide variety of reasons. Some estimates put the area burned in California alone to 1.6 million hectare (ha) per year before Euro-American colonization [31]. However, traditional Indigenous burning practices [32] were considered undesirable and banned from modern forest management practices early in the twentieth century [33] by the US government [34]. Although there has been increasing recognition of the knowledge that can be gained from Indigenous burning practices, current forest conditions and fire suppression policies are impeding efforts to recover Indigenous fire stewardship, associated cultural, social, and spiritual practices, and food security [35, 36].

## Smoke, Human Communities, and Natural Ecosystems

In 2020 alone, over 4.1 million ha burned during summer wildfires in Washington, Oregon, and California [37]. While large fire years can be seen in the historical record throughout the western US [38], large fire events are now responsible for over 90% of burned area and significant increases in fire severity [3, 39]. Given the dramatic rise in western US wildfires and the tremendous amount of smoke emitted by these events, prolonged smoke exposure is impacting communities both near and distant from the actual wildfires [40]. Regional fire events across the western US are expected to increase even under the most optimistic climatic scenarios [3, 5, 41]. Warmer, drier summers — combined with drought and wind events — are catalysts for regional wildfires, smoke, and persistent haze. Of particular concern are synchronous large fires (so-called megafires > 40,000 ha), which are having the most significant ecological, economic, social, and human health impacts within and across regions [42–44].

In western US conifer forests, wildfires generally range from low-severity fires that burn surface fuels below forest canopies to high-severity crown fires that burn both surface fuels and tree crowns [45]. However, many seasonally dry forests are now more susceptible to high-severity fire events due to the legacy of fire exclusion. High-severity crown fires represent a maximum amount of fuel consumption, energy release, and smoke emissions that a fire can generate. In places where organic soils and coarse wood have accumulated under a prolonged period of fire exclusion, smoke production from large, high-severity fires can last for weeks and months [46–48], and can often only be extinguished by a season ending weather event.

Recent wildfire seasons underscore the regional and international smoke impacts on communities [16, 48–50]. Community exposure to smoke is one of the most important public health and health equity considerations in fire management and community planning [51]. Recent long-duration smoke events associated with regional wildfires highlight communities that are particularly susceptible to smoke impacts, including those that are not prepared for extended periods of unhealthy air [8, 52, 53]. Another challenge in the western US is rapid population growth over the last three decades, which has led to drastic land use changes and urban expansion, with particularly rapid growth in fire prone areas of the wildland urban interface (WUI) [54, 55]. As people move from urban areas to suburban and rural communities, more people are directly exposed to prescribed fire and local smoke events [56, 57]. A substantial percentage of the population within California (3.3%), Washington (9.6%), and Oregon (15.9%) lives in very close proximity of seasonally dry forests (Table 1), which is particularly important from a land management perspective.

**Table 1:** Populations living in and near the wildland urban interface (WUI) in the western US. This table displays populations and housing units located in the WUI within and adjacent to seasonally dry coniferous forests (LANDFIRE fire regime groups 1 and 3) in Washington, Oregon, and California. The 1 km buffer is intended as a conservative estimate of proximity to prescribed fire and local smoke events. WUI was defined and calculated based on block level housing units and populations (2010 dataset), and WUI data were obtained from the Silvis Lab at University of Wisconsin (2017 dataset) [58]. For the purposes of this table, WUI intermix, the area where structures and wildland vegetation directly intermingle, and interface, the area where structures are adjacent to the wildland vegetation, were combined into the “WUI” [55]

	In WUI overlapping seasonally dry forests		In WUI + 1 km buffer	
	Population (% of total population)	Housing unit (% of total housing units)	Population (% of total population)	Housing unit (% of total housing units)
*WA*	43,536 (0.6)	22,457 (0.8)	644,947 (9.6)	307,344 (10.7)
*OR*	64,767 (1.7)	29,334 (1.8)	609,964 (15.9)	282,124 (16.9)
*CA*	103,494 (0.3)	56,362 (0.4)	1,230,627 (3.3)	649,875 (4.8)

## Forest Restoration and Fire Management

Forest restoration in seasonally dry pine and mixed-conifer forests aims to modify the current forest fuel structure to reflect conditions supported by more characteristic active fire regimes within a changing climate. Interventions include reducing tree density, favoring larger tree sizes, and fire-tolerant tree species with the goal of promoting resilience to future disturbance and climate change [13, 14, 59]. Increasing the extent of forest restoration treatments is essential to preserve forest ecosystems into the future as the extent and severity of wildfires and other disturbances increase. Historical fire regimes effectively maintained variation in tree cover and species composition at scales ranging from one hectare to hundreds of thousands of hectares [14, 60]. Such variation and its inherent resiliency are what forest restoration ultimately strives to re-create [59]. Although this review is focused on seasonally dry forests, the lessons learned may become more widely applicable as more forests become drier for longer intervals, and therefore more susceptible to a more active fire regime.

Restoration in seasonally dry forests usually involves strategic fuel reduction, and the retention of more fire-resistant tree species [61]. While fuel reduction can involve mechanical or hand thinning of trees and shrubs, it usually requires the application of prescribed fire to effectively moderate future fire severity [62, 63]. Fire is often used as part of a fuel reduction treatment, ranging from the targeted burning of hand or machine piled fuels created during thinning to the planned, intentional application of broadcast prescribed fire across a landscape to consume residual surface fuels, to the management of unplanned lightning ignitions to burn at low-to-moderate severities across a landscape where ecologically appropriate (managed wildfire for resource benefit).

These different fuel reduction techniques — thinning, pile burning, prescribed fire, and managed fire — vary in their effectiveness, duration, expense, and smoke production. Each of these techniques can effectively mitigate subsequent wildfire behavior and lessen smoke production until surface and canopy fuels once again increase [64], which can occur within 10–20 years of implementation [65]. Thus, continued treatment maintenance using prescribed fire or managed wildfire is often the most financially cost-effective approach, particularly if treatments can be used as potential control locations on the landscape to contain larger managed wildfires [2]. A growing body of research on forest restoration in seasonally dry forests concluded that fuel treatments generally require (1) an initial application of fire to reduce subsequent wildfire intensity (summarized in Prichard et al. 2021), and (2) continued burning of increasingly large landscapes to efficiently maintain the reacquired resilience [2, 66, 67]. As such, smoke exposure is an inevitable byproduct of any effective restoration [44]. Understanding and accounting for smoke production versus exposure and its health effects is critical to evaluating the impact that forest restoration and fire management have on public health.

## Affected Populations

Here, vulnerability encompasses a greater likelihood of exposure, a greater sensitivity to the health of well-being impacts of exposure, or greater susceptibility to harm and lack of capacity to cope and adapt [68]. The adaptive capacity of a community is defined by its ability to adjust to potential damage, to take advantage of opportunities, or to respond to consequences [69]. The resilience of a community is defined by its ability to anticipate, absorb, accommodate, or recover from a hazardous event — responding in ways that maintain essential function, identity, and structure, while also maintaining the capacity to adapt, learn, and bounce forward [70, 71]. The building blocks of community resilience include the socioeconomic context, community assets, and social capital, all of which are necessary for broader system resilience and adaptation [71–73]. Examples of vulnerability, adaptive capacity, and resilience metrics available for western US are shown in Supplemental Table 3.

To integrate equity into forest management, each community’s vulnerability, adaptive capacity, and resilience must be taken into consideration. One pathway to equity can be achieved through community-tailored, culturally informed smoke management practices, achieved through early and continuous consultation with impacted communities. Another path toward equity is through policy, in which researchers, policymakers, practitioners, and managers purposefully integrate the principles of EJ across all aspects of their work. Formal definitions of EJ can provide practitioners with an initial lens. As example, Washington State recently passed the Healthy Environment for All (HEAL) Act, which establishes a formal definition of environmental justice in Washington and expands on the US Environmental Protection Agency’s (EPA) definition (underlined): “Environmental justice means the fair treatment and meaningful involvement of all people regardless of race, color, national origin, or income with respect to the development, implementation, and enforcement of environmental laws, rules, and policies. Environmental justice includes addressing disproportionate environmental health impacts in all laws, rules, and policies with environmental impacts by prioritizing vulnerable populations and overburdened communities, the equitable distribution of resources and benefits, and eliminating harm” [74]. In the context of wildfires, EJ would focus mitigation on those most impacted by smoke exposure, seek to address past and current damages, and inspire structural change to prevent future harm. The ethical imperative is to prevent disproportionate burden of smoke on vulnerable communities.

Smoke from wildland fires has varied effects on human health depending on the populations exposed. Some of the populations vulnerable to the health impacts of smoke are children, the elderly, and individuals with pre-existing health conditions. Populations more vulnerable to smoke exposure include people in low-income communities, people living in homes with poor air filtration systems, people experiencing homelessness, and workers in high-exposure occupations. Communities that lack resources to plan for and mitigate long-duration smoke events are more susceptible to experiencing health impacts [75, 76]. Where someone lives, works, studies, and plays all determine their exposure to smoke (see description of *exposure* in the following section). Among smoke-exposed communities, those that are predominately Black, Latinx, and Native American may experience 50% greater risk of smoke exposure based on systemic and structural inequities [75]. Additionally, several recent analyses observed that communities that are more frequently adjacent to prescribed burning are also those with socio-demographic and health vulnerabilities [77, 78]. These and other factors give every community a distinct susceptibility profile. However, current risk identification strategies based on smoke exposure levels alone do not account for these differences [40].

During a large smoke event, large populations can be exposed to the plume. However, exposure is likely higher for those who spend more time working outdoors without respiratory protection, have tasks requiring higher levels of physical exertion, and experience little control over their work environments due to workplace conditions or power dynamics [79–82]. Outdoor working populations that may be more frequently exposed to smoke and often work with exertion include agricultural and forestry workers [79, 81]; wildland firefighters; construction, grounds maintenance, and landscaping; and transportation, utility, and recreation workers [83] (see Supplemental Text 2 for an exhaustive list). Some employers use air quality index (AQI) classifications to inform outdoor work decisions, but in most states, this practice is voluntary. The AQI for particulate matter converts 24-h averages of particulate matter (PM_2.5_) in μg/m^3^ into categories that correspond to levels of health concern [84]. The higher the AQI value, the greater the level of air pollution and health concern. For example, if the 24-h average for PM_2.5_ is under 12 μg/m^3^, the AQI will be between 0 and 50, or “good.” In California, outdoor protection from wildfire smoke focuses on a fine particulate matter (PM_2.5_) AQI that is above 151, which corresponds to “unhealthy for all people” [85].

Importantly, some populations are at particularly high risk of experiencing health impacts of wildfire smoke. For individuals with COPD or other respiratory conditions, exposure to smoke can be life-threatening [86]. Pregnant women are also an at-risk population, as in utero exposure has been positively correlated with pre-term birth and decreased birthweight [87].

The health burden of air pollution is rising with the increasing severity and frequency of fires. Although exposure to wildfire smoke has become more and more prevalent, populations in these risk categories are often not aware of the health impacts of smoke exposure. Healthcare providers and community health workers should include precautionary warnings and recommendations for how to stay safe during wildfire season during check-ups with at-risk patients [88]. Investment in culturally appropriate information sharing with at-risk populations could enhance equity to smoke exposure outcomes and an individual’s ability to stay safe during fire season.

## Health Effects of Wildfire Smoke Exposure

Epidemiologic studies have consistently shown an association between exposure to wildfire smoke and increased risk of adverse respiratory health outcomes and all-cause mortality [52, 89]. A growing number of studies indicate that wildfire smoke exposure may also increase risk of adverse birth outcomes and cardiovascular events [8, 9, 87, 90]. A recent study found that exposure to smoke during a severe wildfire event led to a 70% increase in out-of-hospital cardiac arrests, with larger risk among low socioeconomic groups [91]. Populations with existing vascular disease, heart failure, and/or diabetes mellitus are especially vulnerable to experiencing the health impacts of wildfire smoke [92, 93]. Overall, the smoke epidemiology literature is consistent with the much larger literature on adverse health effects of urban and traffic-related air pollution [93, 94].

Most recently, studies examined whether exposure to wildfire smoke can increase susceptibility to developing and dying from COVID-19 [95–98]. Though limitations of research linking COVID-19 to air pollution are well-described [99], they add to a growing number of studies relating adverse COVID-19 outcomes to outdoor and urban air pollution [100–102]. For example, a recent study in China assessed the risk of COVID-19 infection related to short-term air pollution exposure and found significant positive correlations with increased levels of PM_2.5_ [103]. Another study in the United States analyzed county level data and found an 11% increase in the COVID-19 fatality rate for every 1 μg/m^3^ increase in annual PM_2.5_ exposure [104]. In a hypothetical thought scenario, Henderson [96] estimated that, depending on timing, a moderate wildfire smoke event has the potential to increase the impact of a COVID-19 outbreak by more than 10%.

Many epidemiologic studies have attempted to characterize the health effects associated with exposure to wildfire smoke for populations. A review by Reid et al. [52] found acute outcomes (measured as increased hospitalizations and emergency room visits) during and after major smoke events are associated with admissions for exacerbation of an existing respiratory condition such as asthma or COPD. In a more recent meta-analysis by Kondo et al. [105], authors outlined the heterogeneity of responses to smoke exposure within a population. As mentioned in the previous section on affected populations, sex, age, race, income, education, housing, access to healthcare, and many other factors all have an effect on health risks to smoke exposure. Children are at particular risk, which is well-documented in studies demonstrating the health effects of PM_2.5_, including but not limited to respiratory diseases such as asthma [9, 106], lung development [107], and lung function [108].

The long-term respiratory health effects of severe or repeated exposures to wildfire smoke are largely unknown [109]. However, both short- and long-term occupational exposures to smoke are well-documented among wildland firefighters [110–112]. Similar to other comparisons between occupational and ambient exposures, wildland firefighters typically have far greater exposure than the general public due to factors such as closer proximity to the source, different compositional exposure, and longer periods of exposure [113, 114]. Epidemiologic studies of repeated exposures among firefighters suggest that cumulative exposure to smoke throughout the wildfire season increases airway inflammation and decreases lung function [110, 113, 115, 116]. However, results are inconclusive as to whether this decreased function is sustained or returns to baseline in the off-season [113]. Additionally, wildland firefighters are more likely to suffer from cardiovascular outcomes, including hypertension, and have a higher CVD mortality than non-firefighters in a similar age group [117, 118].

Distinguishing the health effects from smoke exposure during repeated prescribed burning versus a multi-week single wildfire is complex, especially when considering the existing vulnerabilities of disproportionately affected populations. To date, there has been little research on the trade-offs between wildfires and dry forest restoration practices on the pollutants emitted [119] and subsequent health effects. Very few studies have examined health effects of exposure to PM_2.5_ specific to prescribed fire smoke [112, 120–122]. A preliminary study from Prunicki et al. [122] collected data from children in Fresno, California, who were living within a 70-mile range of a wildfire, a prescribed fire, or no fire during the spring and fall of 2015. Immune profiles indicated a trend toward increased inflammatory markers in children exposed to the wildfire, and health questionnaires showed worsened outcomes such as wheezing in those without asthma and exacerbations in those with asthma. These outcomes, however, were not significantly different from children exposed to prescribed fire smoke, which, although less than the wildfire group, also demonstrated increased inflammation and asthmatic symptoms. Another study in Australia examining asthma symptoms in both children and adults suggested that the lower and shorter exposures to PM associated with prescribed burning led to less severe health effects than exposure to wildfire smoke [123]. In these studies that compare wild and prescribed fires, there is a lack of consistent study objectives and methodologies to make adequate comparisons between the two scenarios [9, 112, 122].

## Exposures and Co-exposures

An exposure pathway varies over space and time but is defined by the course that smoke travels from its source (fire), through a medium (air), to a receptor (human inhalation). Assessment of individual or population exposure to smoke is complicated by several factors, including that it is often based on retrospective models using different techniques [124]. Additionally, smoke from biomass combustion is a complex mixture of thousands of chemicals, a composition that changes dramatically when fires move into the WUI and consume anthropogenic materials that release other toxic chemicals that do not exist in natural ecosystems [125]. Smoke particles can travel great distances and age as they react with sunlight and other chemicals, which may increase toxicity [126]. However, a larger dose of less toxic particles may still be more harmful than a smaller dose of more toxic particles [127].

Many pollutants in wildfire smoke are already regulated by the EPA as pollutants (e.g., PM_2.5_, CO, NO_X_, phenols, cresols, acrolein, and acetaldehyde) — though not regulated when wildland fire smoke is the source. At least five chemical groups in smoke have been classified as human carcinogens by the International Agency for Research on Cancer (IARC) [114]. Firefighters during wildfires or prescribed burns are exposed to significant levels of CO, formaldehyde, acrolein, and respirable PM_2.5_ [128, 129]. Environmental exposures of wildfire smoke can reach dangerous levels of PM_2.5_ and contain high concentrations of black carbon, Cu, Zn, Pb, Sn, and other metals [130]. Our understanding of exposure profiles for wildfire versus prescribed fire smoke is limited but will continue to improve through source apportionment of smoke composition as a function of time, location, fuel source, and combustion phase during fires.

Wildfires and prescribed fires both release large amounts of pollutants in the atmosphere and are therefore important contributors to ambient air pollution [131]. The composition, volume, and dispersion of smoke plumes are dependent on numerous factors, including fuel type, total fuel amount, fuel moisture, area burned, fireline intensity, fire duration, and fire weather [11, 132]. As a result, the spatial and temporal patterns of wildfire and prescribed fire smoke exposures vary. Wildfires often occur under hot, dry, windy conditions, which can increase plume rise and transport, driving longer smoke exposures across larger geographic areas [11, 133, 134]. On the other hand, prescribed burns are generally smaller, lower intensity fires that usually take place under meteorological conditions that are less conducive to long-range atmospheric smoke transport (i.e., low wind speed, low temperature, and high relative humidity), suggesting that prescribed smoke exposures may affect populations located closer to the burn areas, for a shorter duration, and with lower PM_2.5_ emissions [135]. However, as mentioned in the previous sections, recent analyses have also shown that often communities that are more frequently adjacent to prescribed burning are also those with socio-demographic and health vulnerabilities, thus altering the impact of prescribed fire smoke [77, 78].

**Fig. 2:**
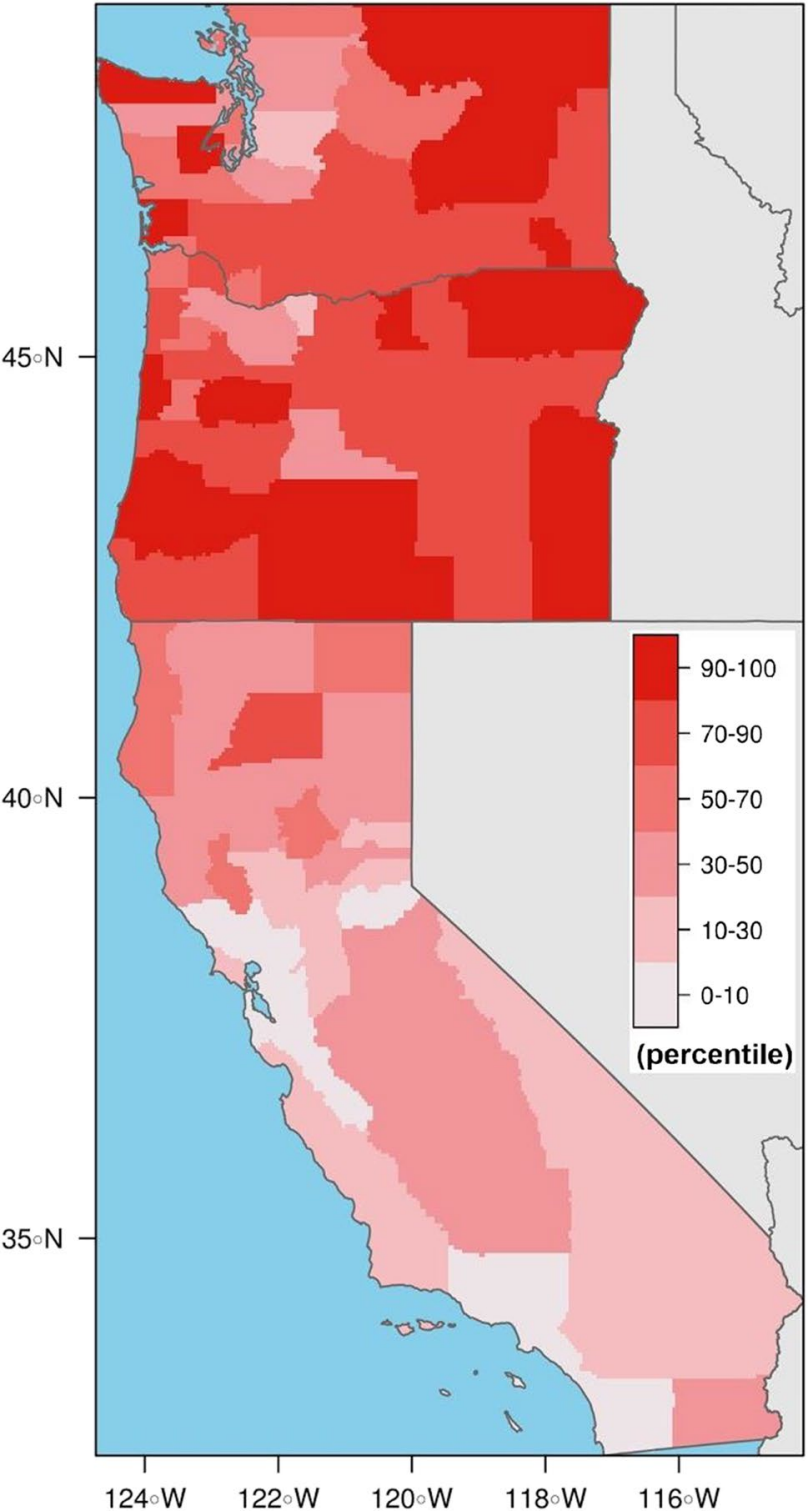
Populations vulnerable to the health risks of smoke exposure. The Community Health Vulnerability Index (CHVI) is designed to capture community vulnerability to wildfire smoke based on factors known to increase the risk of health effects from airborne pollutants (15 parameters described in Supplemental Text 1). Percentiles by county are adapted from Rappold et al. [40]. Higher percentiles indicate more vulnerable areas

The health risk of co-exposures to fire smoke and other pollutants increases as smoke drifts into more populated and polluted areas. In recent years, regions in the western US that have been overwhelmed with wildfire smoke during the summer months are some of the same regions already struggling with high levels of urban or agricultural pollution [48, 81]. Additionally, many of these regions fall into the 70–100th percentile on the Community Health Vulnerability Index (CHVI) developed by Rappold et al. [40] (Fig. 2). The five components of the CHVI are (1) economic deprivation, (2) population of 65 years and older, (3) chronic adult respiratory conditions (COPD and asthma), (4) pre-existing conditions linked to hypertension, obesity, and diabetes, and (5) pediatric asthma, and explained 84% of variance (SI Table S2). Each state has its own smoke management plans and regulations that limit increased use of prescribed burning based on several factors including current air quality, wind direction, and regional haze regulations, which can become the limiting factor for burn authorizations [136]. States are responsible for adhering to the 1999 EPA Regional Haze Rule [125] which prioritizes airshed visibility, and to the National Ambient Air Quality Standards (NAAQS) which cover the six criteria pollutants (CO, Pb, NO_x_, O_3_, PM, and SO_2_). These regulations *limit* co-exposures; however, decision-making around prescribed burning is usually based solely on levels of PM_2.5_ and does not include considerations for the health risk profile of surrounding communities.

## Discussion: Transdisciplinary Consensus, an Interdisciplinary Approach

The above sections point to several areas where ecological and human health goals can be advanced through forest restoration. A first step is to identify and articulate areas of agreed concern and advancement. As a transdisciplinary group of scientists, practitioners, and managers specializing in areas of forest and fire ecology, fire safety, air quality, healthcare, and public health, we have come to a consensus on the following statements and recommendations as a guide to better integrate forest and human health. Here, we articulate consensus statements which are followed with further descriptions and motivations for each.

1. We recognize the need to listen to and integrate a diversity of perspectives, in particular those embodied by Indigenous peoples who have successfully used fire as an ecological tool for thousands of years.

Prescribed and managed fire are critical components of many Indigenous efforts to revitalize and maintain fire cultures and stewardship of traditional lands (for example, see the Indigenous Peoples Burning Network [137]). Indigenous populations in the western US were using fire as a landscape and cultural resource management tool for millennia prior to colonization by Euro-Americans [138, 139]. However, the potential ecological impacts and social benefits of this extensive environmental modification were not well recognized by western scientists until recently [140]. Information about Indigenous burning at large scales is part of many Indigenous cultural narratives [139], and research documenting Indigenous fire use in land stewardship practices is increasingly documented (e.g., see [35], and references therein). There is a need to understand and respect the stories that remain, and to integrate Indigenous expertise into current stewardship practices. There is also a need to center the leadership and perspectives of tribal nations and communities. For example, this type of integration is beginning in California with the Karuk and Yurok tribes as they advocate for expansion of prescribed and cultural burning [141].

2. Prescribed fires in addition to managed fires for resource benefit are both necessary management techniques to keep forests resilient and to lessen the negative ecological and public health impacts of wildfires.

Severe wildfires are burning western US landscapes at an accelerating pace and scale. Climate change is exacerbating the acceleration, creating an added urgency to this environmental and social crisis. Along with direct impacts of fire, smoke is a critical public health issue for all fires, prescribed or otherwise; thus, we must strive toward a “least smoke alternative” over the coming decades. There is no future without fire and smoke, but with aggressive, restoration- and adaptation-focused forest management including prescribed fire, managed wildfire, and mechanical thinning, we can significantly improve the outcome from continued suppression in a warming and drying climate. *Large scale interventions are needed to mitigate both the human health and ecological outcomes from increasingly large, severe wildfires*. Prescribed and managed fire can reduce the severity and extent [25] of subsequent wildfires [142, 143], emissions from prescribed and managed fires are much lower, and their smoke dispersion and direction can be more effectively managed, compared with megafires [11]. However, empirical evidence is thin for experimentally testing prescribed and managed fire effects at very large scales (1,000’s to 100,000’s of ha) [144], but see Prichard et al. [145] to highlight important new directions on this particular question. Additionally, increasing the use of prescribed and managed fire will require coordination across multiple sectors [136]. We have the potential to reduce hazards by shifting from a policy that emphasizes fire suppression to one that favors intentionally creating fire-adapted forests and communities, thereby increasing the likelihood of reducing the severity of peak-season fires.

3. Certain regions of the western US will experience more smoke days with heightened use of prescribed and managed fire; however, we expect the impacts of smoke exposure to be reduced over the long term in comparison with untreated land burned by wildfires. With these techniques, exposure in affected communities can be planned and lessened.

Collaborative planning is essential to mitigate smoke exposure. For example, the California Air Resources Board (CARB) designates days within individual air basins as Permissive-Burn, Marginal Burn, or No-Burn based on predicted air pollution levels and meteorological conditions related to smoke transport [146]. Prescribed fire is prohibited on No-Burn days but may be permitted on Permissive-Burn days or Marginal Burn days, with fewer acres permitted on the latter. Following the 2007 Angora Fire in the Lake Tahoe Basin, CARB adjusted the regional criteria used for burn day designation, giving mixing height and transport winds greater importance and allowing for a greater number of burn days in the region, while still reducing smoke exposure [147]. *Land managers and air quality regulators are increasingly working together to achieve both forest health and public health objectives*.

Although choice of burn days based on air quality standards limits public health impacts of smoke from prescribed fire, it also significantly reduces opportunities to conduct burns. The number of desirable low impact smoke days is in steep decline and will continue to decline. Changes to air quality regulations have been successful in other regions across the United States, including the North American tallgrass prairie and the Kansas Flint Hills, where *expansion of the burn window increased flexibility to help manage smoke exposure* to downwind cities [148, 149]. Researchers in this region found that burning outside the late spring burn window not only improves local ecology but benefits humans as well [148]. These examples demonstrate how more collaborative and intentional planning of prescribed burns can work to minimize community smoke exposure.

4. No degree of smoke exposure is without risk. However, additional investment in advance preparation for affected populations can lower associated health risks. A smoke-resilient community is resilient to smoke from any type of fire.

Mitigation of smoke exposure can also occur within affected communities. In a recent workshop hosted for practitioners and managers in the forest, fire, air quality, and public health disciplines in Washington State by this working group, there was consensus across disciplines that increasing awareness of the health impacts of smoke and working toward smoke-ready — not just fire-ready — communities would significantly improve health outcomes. Although more work is needed, *there are currently many strategies and tools that have been developed to protect public health and increase community resilience to smoke* [150–153]. Strategies identified during this engagement workshop and in the literature include but are not limited to emergency notifications before the smoke arrives to warn that air quality may reach unhealthy levels, sheltering in place at home, access to community cleaner air shelters [151, 153, 154], paid leave for workers during smoke events, and providing the public with information on effective use of masks [152]. For workers exposed to fire smoke, *policy changes can also help to lessen exposure*, where health protections can be enforced through federal or state occupational health and safety regulations [83, 155]. For example, California has enacted regulation to protect employees exposed to wildfire smoke that increases communication and training on protective measures for hazardous smoke exposure [156]. Washington and Oregon have issued public notices for similar rulemaking to protect exposed workers [157, 158].

5. We must work to promote both equity in process (e.g., who has a say in decision-making) and equity in outcomes (e.g., who gets exposed to the smoke) within those communities and populations experiencing disproportionate impacts from smoke.

It is essential that risk communication around the health effects of wildfire smoke and strategies for how to reduce exposure are effectively disseminated to all affected populations [159–161]. Communication related to proper mask use, and the distinction between respirators and other masks, was particularly relevant in 2020 to minimize COVID-19 and wildfire smoke co-exposures among agricultural communities. Community-based organizations and local health experts co-developed bilingual Spanish-English public service announcements, radio spots, webpages, social media messages, infographics, and training material about recent public health emergencies for specific communities [162]. *Trusted content and cultural and language experts must be involved* to ensure such health messages are accessed, shared, and understood. This type of outreach and messaging is an example of one of the ways equity in process can be promoted. Again, we must also ensure that these messages are disseminated to all communities affected by smoke and other airborne pollutants [160]. For example, Ojerio [163] found that wildfire mitigation strategies, such as Community Wildfire Protection Plans, target areas of high biophysical risk without taking vulnerability, adaptive capacity, or resilience into account. In order for these types of programs to effectively demonstrate equitable solutions, *the vulnerability of affected populations must be taken into consideration*. As previously mentioned in the “*Affected Populations*” section, this is beginning to be addressed in the Washington State HEAL Act, which defines EJ in state law and outlines how the needs of communities disproportionately affected by pollution must be considered by decision-makers.

6. We are missing opportunities for positive impact by working as separate disciplines. We recommend that further and intentional integration of forest/fire and health disciplines (including the practitioners, tools, and resources) needs to occur to lessen the human health effects of smoke exposure due to prescribed and managed fires.

Opportunities and roadblocks for collaborating across disciplines are widely recognized [12]. As a working group, we have learned what motivates each of our disciplines and have agreed that integrating our knowledge and resources is mutually beneficial. This type of transdisciplinary collaboration has been increasing, especially in the area of climate research [150]. Recently, groups such as the Cascadia Wildfire and Urban Smoke Working Group; the Central Oregon Prescribed Fire, Public Health, and Smoke Collaborative; the National Academy of Sciences; and the EPA have brought disciplines together to specifically address wildfire smoke. These groups and others are educating across disciplines through workshops and webinar series, as well as through discussions within intentionally designed working groups. But *more work is needed to overcome roadblocks to collaborative work*, including removal of funding constraints and provision of institutional and professional incentives and rewards. Mechanisms, such as the Science for Nature and People Partnership and the National Socio-Environmental Synthesis Center [164, 165], provide much needed assistance and resources to bring together practitioners, researchers, and decision-makers from various disciplines and sectors. Through this type of deliberate support, intentional discussions and ideas that speak to diverse stakeholders can emerge for immediate action.

Federal policy over the past few decades has largely been supportive of forest restoration and climate adaptation, and there are increasing avenues for community engagement and adaptation to wildfires and smoke. The forestry provisions of the Build Back Better Bill allocate approximately $27B for federal, state, and tribal forests, with a major chunk of that going to wildfire resilience and prevention [166]. During 2021, Oregon passed SB 762, a $220M bill to create fire-adapted communities, increase landscape resiliency, and improve safe and effective wildfire response [167]. This federal and state level attention provides an opportunity to move forward as a transdisciplinary community. However, barriers to broad implementation still vary across different states and local communities [168, 169]. Supplemental Text 3 is a report detailing policy leverage points for integrating public health and increased use of prescribed burning in Washington, Oregon, and California. This report can be used as a tool by both policymakers and practitioners to understand barriers and facilitators to the application of a public health perspective to prescribed and managed fire.

A wide range of organizations and tools already exist that could help to further facilitate transdisciplinary integration. A comprehensive list of these tools and organizations, with specific examples for a few that could be utilized across disciplines, can be found in Table S4.

## Conclusion

As part of this interdisciplinary dialogue, we asked: (1) what are the public health outcomes of status quo fire and smoke management in the western US, and how might smoke emissions and associated health outcomes be reduced by alternative management scenarios?; and (2) how might public health be most effectively and equitably incorporated into the management of western US forest landscapes historically maintained by frequent fire? The status quo of fire and current smoke management strategies combined with climate change have led to increasingly longer fire seasons, larger and more severe fires, and thus longer smoke seasons with greater human exposure to smoke. Smoke exposure is associated with human health effects, including exacerbation of existing respiratory diseases such as asthma and COPD and other effects described in the section “*Health Effects of Wildfire Smoke Exposure*.” As stated in consensus statements 2–5, if managers and communities work together on the increased use of prescribed and managed fire to combat this climate and public health crisis, communities that are impacted by smoke can increase efforts to plan and prepare for exposures during and beyond fire season. As stated in consensus statement six, to integrate public health into the management of western US forest landscapes, support and resources are needed to foster key collaborations between practitioners, researchers, and decision-makers from various disciplines and sectors.

Wildfire is an inevitable and an essential component of many ecosystems. Nonetheless, recent fires have had significant adverse impacts on forest and human health, and these impacts are expected to increase as area burned and fire severity increase in the coming years. Each wildfire season, these adverse impacts receive considerable attention from both the public and policymakers. However, if any meaningful changes are to be made, more consistent awareness and action are needed.

Researchers, practitioners, and stakeholders in the field of forest and fire management are increasingly recognizing the need to incorporate human health, health equity, and EJ considerations into ecological forest management and planning. Health and equity professionals are increasingly interested in how forest and fire management can be used as a tool for reducing the human health risks and inequities from wildfire. Our review applied a transdisciplinary perspective to the pressing issue of ecological and human health effects from wildfires. Through a review of the existing literature, we sought to identify integrated perspectives to advance this area of practice. We identified six consensus statements, as well as areas for future work.

There are still many unanswered questions. From a public health perspective, the long-term health effects of prolonged and repeated exposures to smoke are currently understudied though this research space is growing. Still lacking is adequate comparative evidence examining the health effects of smoke from different types of fires (e.g., wild versus prescribed under varied fuel and weather conditions). There are many existing resources to help communities become more resilient to the impacts of fire and smoke, but little is known about the effectiveness of the different strategies. From the forest and fire perspective, more insight is needed into the trade-offs between forest and fire management strategies and air quality. Existing barriers to increasing the use of prescribed burns are often political and based on smoke aversion.

Although more work is needed, this integration of knowledge and resources, and education between disciplines, was an essential first step. By discussing our areas of expertise on the shared topic of wildfires, we were able to generate new, integrated ideas and come to shared consensus, articulated in the synthesis above. Statements that seemed straightforward to one discipline in the beginning (for example, *fire is inevitable*, or *all smoke exposure is harmful to health*) were not readily accepted by all. It was a discussion of trade-offs, shared and not shared goals, and an overall desire to learn and contribute that moved the conversation forward. Through this understanding, and integration of our perspectives, we can move from conversation into action.

## Supplementary Information

Below is the link to the electronic supplementary material.

Supplementary file1 (DOCX 1028 kb)

## Data Availability

All data are available in the main text or the supplementary materials.
