# Supplementary material for: Wildfire, Smoke Exposure, Human Health, and Environmental Justice Need to be Integrated into Forest Restoration and Management
Source: Curr Environ Health Rep. 2022 May 7;9(3):366–85. doi: 10.1007/s40572-022-00355-7 (PMC9076366; doi:10.1007/s40572-022-00355-7)
Supplement: Supplementary file 1 — Supplementary file1 (DOCX 1028 kb) [file 40572_2022_355_MOESM1_ESM.docx]

Supplementary Text 1: Methodology for Main Manuscript Figures

**Figure 1**

Figure 1 C was developed first to categorize the fire regimes in WA, OR and CA. This map was then utilized to create Figure 1 A and B. We explain the methodology in this order.

**Figure 1. C**

We downloaded 30 m resolution 2014 fire regime data and a separate existing vegetation data (LF 1.4.0 version) from https://www.landfire.gov. We then reclassified the fire regime data into 6 groups: fire regime 1, 2, 3, 4, 5, and other lands which combine all other fire-regime-irrelevant categories (water/snow/ice/barren/sparsely vegetated land). We also recategorized the existing vegetation data into 4 groups: coniferous forest land, developed land, agricultural land, and other lands (water/snow/ice/barren/sparsely vegetated land). Since the existing vegetation data have 838 types of vegetation, we used Society of American Foresters-Society for Range Management (SAF-SRM) cover type and Landfire Existing Vegetation Type (EVT) physiognomy to define developed land (SAF-SRM = “LF 20: Developed”), agricultural land (SAF-SRM = “LF 80: Agriculture”), and coniferous forest land (EVT_PHYS = “Conifer” or “Conifer-Hardwood” or “Hardwood-Conifer”). By default, all lands other than these 4 categories within the existing vegetation dataset are “non-coniferous”.

Next, the two reclassified data sets were incorporated and reclassified again into 10 categories: 1) developed land, 2) agricultural land, 3) coniferous fire regime 1, 4) non-coniferous fire regime 1, 5) fire regime 2, 6) coniferous fire regime 3, 7) non-coniferous fire regime 3, 8) fire-regime 4, 9) fire regime 5, and other lands (water/snow/ice/barren/sparsely vegetated land). In this process, we combined fire regimes 1 and 3 with coniferous forest vegetation cover to reclassified them into coniferous fire regime (areas satisfying both fire regime and coniferous forest) and non-coniferous fire regime (areas only satisfying fire regime).

Finally, we simplified 10 categories into 4 categories: 1) developed and agricultural land, 2) coniferous fire regime 1 and 3, referred to as “seasonally dry forests” 3) other wildland vegetation, and 4) water / snow / ice / barren / sparsely vegetated. This process is illustrated in Supplemental Figure 1.


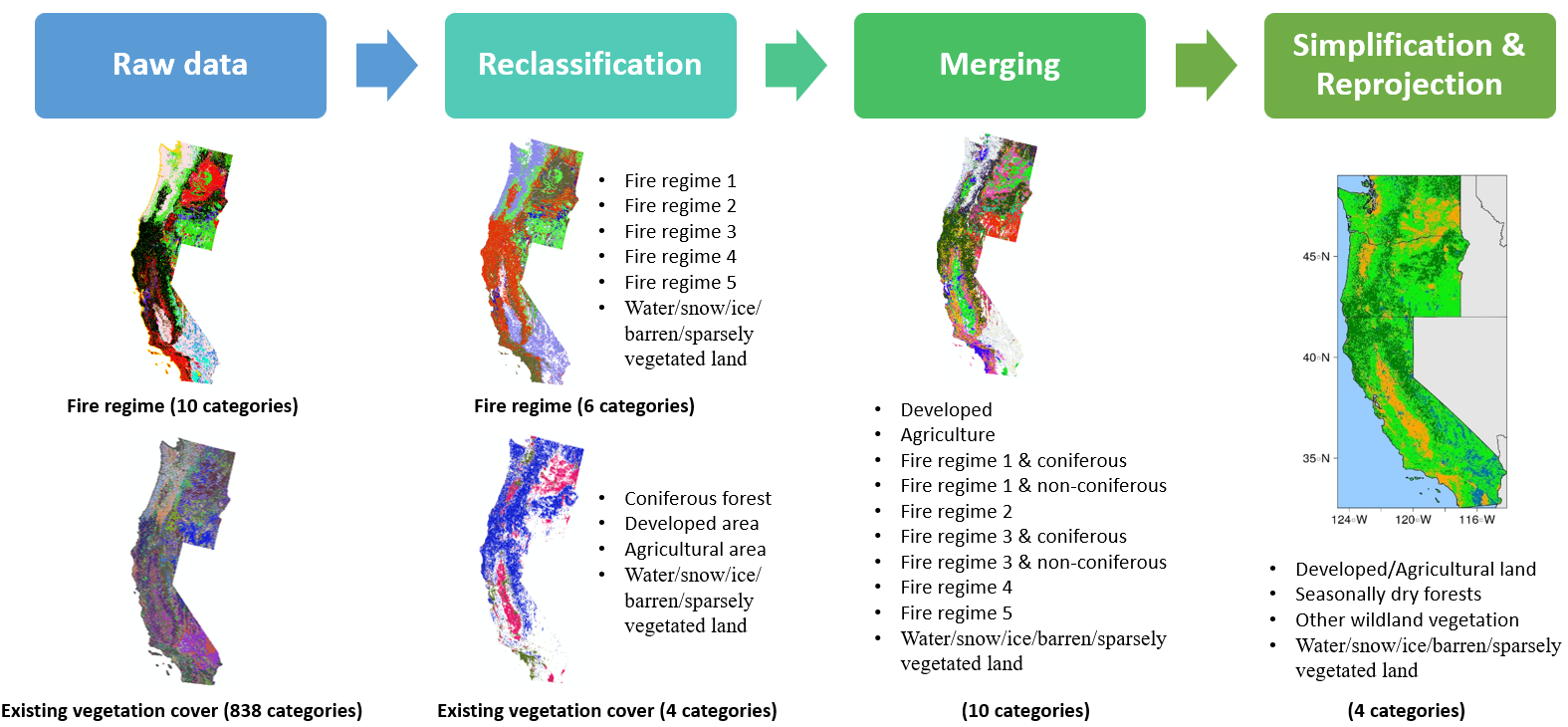


Fig. S1: Schematic process for Figure 1.C

**Figure 1. A**

The Fire Weather Index (FWI) using the Canadian Forest Fire Danger Rating system was calculated with gridMET at a 1/24th degree spatial resolution during 1979–2020 (*145*). We employed a modified approach that uses daily mean wind speed, accumulated precipitation, maximum temperature, and minimum relative humidity, rather than the standard procedure that is based on local noon observations. More details on the method can be found from Abatzoglou et al (2020) (*146*). Daily gridded FWI during 1979–2020 are available at https://climate.northwestknowledge.net/ACSL/GRL/

**Figure1. B**

We downloaded maximum temperature data from http://www.climatologylab.org/gridmet. Since the reclassified fire regime data (Figure1.C, 30m) and the temperature data (4km) have different resolution, we first calculated the centroids of our research interest pixels (i.e. seasonally dry forests - coniferous forest in fire regime 1 and 3) from the reclassified fire regime data. We then extracted and averaged the maximum temperature of the centroids by year and state. This process is illustrated in Supplemental Figure 2.


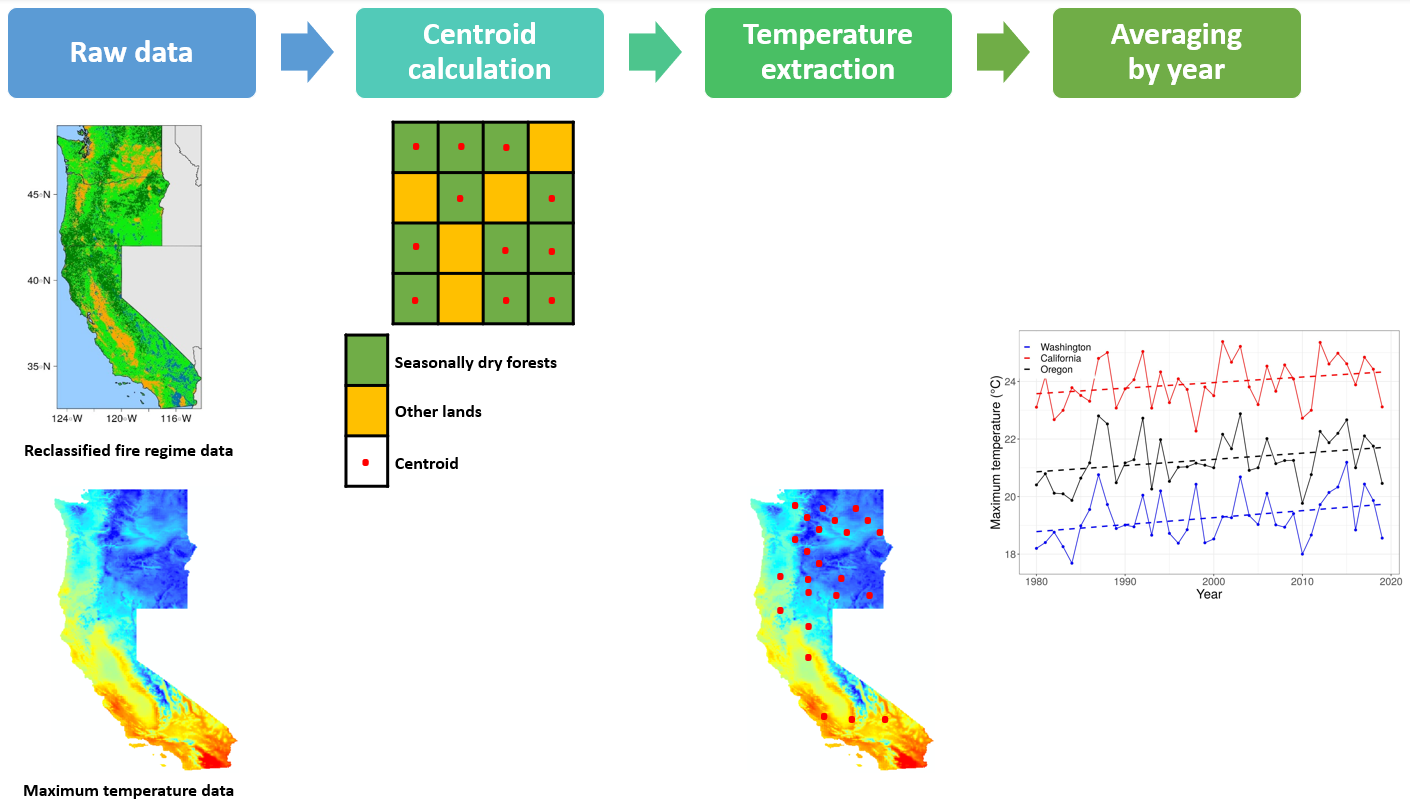


Fig. S2: Schematic process for Figure 1.

**Table 1**

**Columns 2 and 3**

The Silvis Lab at University of Wisconsin (http://silvis.forest.wisc.edu/data/wui-change/) provided WUI (intermix and interface), population, and housing unit data at the census block level. Depending on the type of data, we used a different approach to downscale these data sets. For the population and housing unit data, we evenly divided the census block population and housing unit data by the number of pixels within the block, assuming that they are uniformly distributed across the census block. We, then, assigned the divided value to each pixel. During rasterization, we lost about 0.10 percent of the total population (CA:0.12; WA:0.07; OR:0.11) and housing units (CA:0.14; WA:0.07; OR:0.12). For the WUI data, we assigned the census block’s WUI information (i.e. WUI interface, WUI intermix, or non-WUI) to all pixels within the census block. Through this, we downscaled these data to 30 m resolution data sets to combine with the reclassified fire data (Figure1.C). We then removed seasonally dry coniferous forests in fire regime 1 and 3 smaller than 5 ha. After intersecting the WUI and coniferous forest in fire regime 1 and 3 larger than 5 ha, we were finally able to calculate the total number of population and housing units in WUI overlapping frequent fire forests areas by state. This process is illustrated in Supplemental Figure 3.


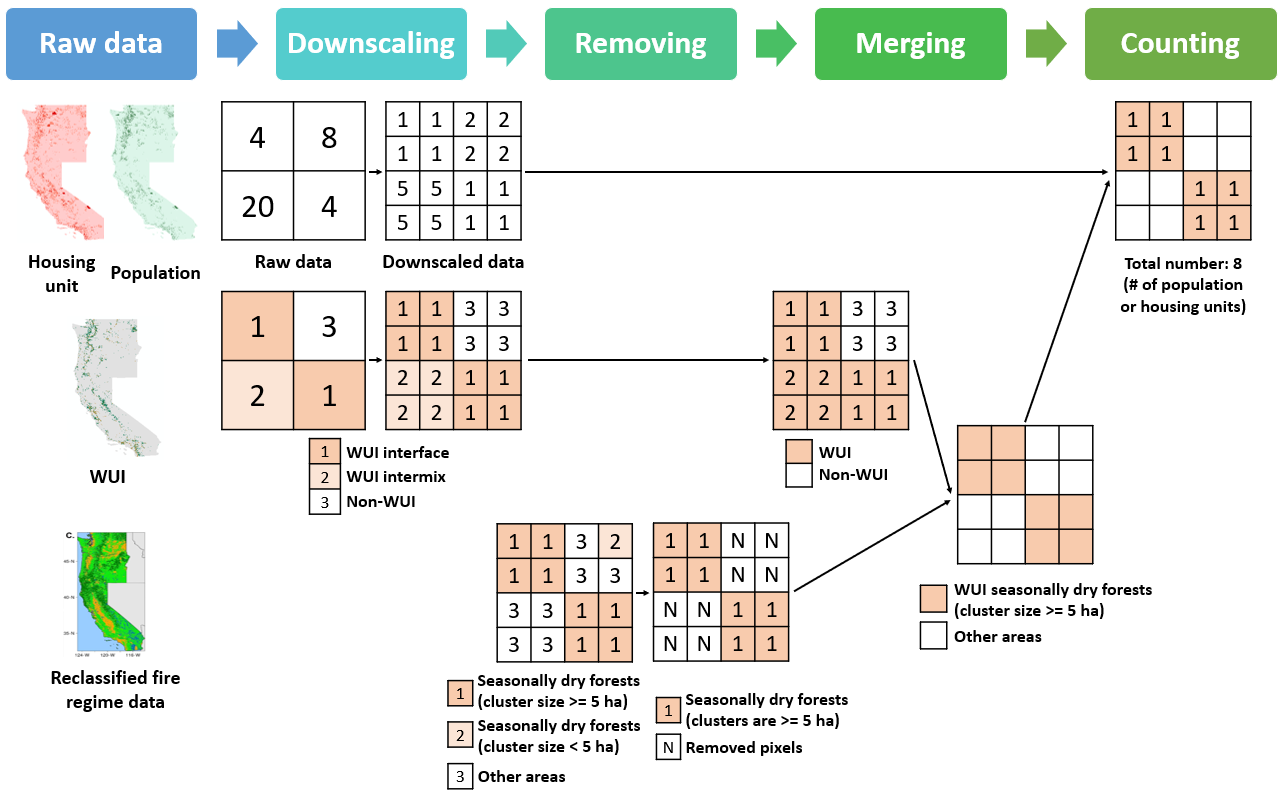


Fig. S3: Schematic process for Table 1 columns 2 and 3

**Columns 4 and 5**

We followed a similar method as in columns 2 and 3. Unlike for columns 2 and 3, we made 1 km buffered zones from the coniferous forest in fire regime 1 and 3 larger than 5 ha to measure the percentage of total population who live within the WUI in very close proximity of frequent fire conifer forests. This process is illustrated in Supplemental Figure 4. Because our housing dataset only includes housing classified as WUI, our buffering process does not account for housing within 1 km of frequent fire conifer forests that is not classified as WUI (e.g. “Non-WUI” pixel class 3 in Supplemental Figure 4). Thus, our population estimates in Table 1 are underestimates of the total population living within 1 km of frequent fire conifer forests.


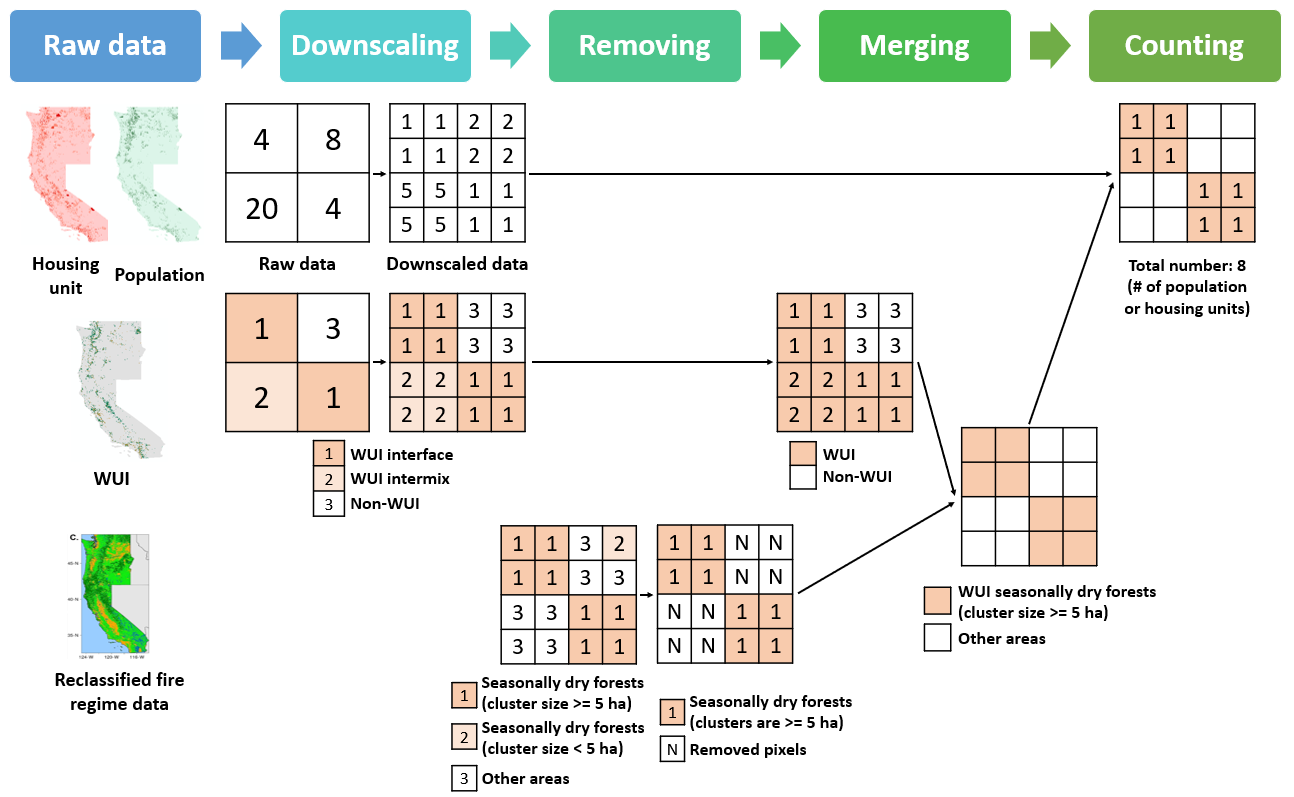
 **Fig. S4: Schematic process for Table 1 columns 4 and 5 (See description in Supplemental text 1.**

Figure 2

We calculated the Community Health Vulnerability Index (CHVI) based on Rappold et al. (2017)’s methodology (*40*). We first collected 15 types of county-level socioeconomic and medical variables (adding three additional variables – marked with an asterisk (*) in Supplemental Table 1 – from Rappold’s 12 variables) from 2019 U.S. Census Bureau American Community Survey (ACS) 5-year estimates, American Lung Association (ALA) 2020 report, and CDC Behavioral Risk Factor Surveillance System 2012 (Supplemental Table 1). After standardizing all variables, we employed Varimax-rotated principal component analysis to reduce the number of variables. The first five principal components, explaining 89% of the total variance, were individually assigned quintile scores from 1 (the least vulnerable) to 5 (the most vulnerable). Then, quintile ranks for each principal component were added together to make the overall CHVI. More detailed information on methodology can be found in Rappold et al. (2017) (*40*).

Table S1: List of data sources for Figure 2.A CHVI. (See description in Supplemental text 1.)

| **Source** | **Variables (n=15)** |
| --- | --- |
| 2019 U.S. Census Bureau American Community Survey (ACS) 5-year estimates | > 65 years |
|  | Poverty (individual) |
|  | Poverty (family) |
|  | With high school education or more |
|  | Median income |
|  | Employment rate |
| American Lung Association (ALA) 2020 report  (https://www.lung.org/research/sota/city-rankings/states) | COPD rate |
|  | Adult asthma rate |
|  | Pediatric asthma rate |
|  | Cardiovascular disease rate * |
|  | Ever smoker rate * |
|  | Non-white rate * |
|  | Hypertension rate |
| CDC Behavioral Risk Factor Surveillance System 2012  (https://www.cdc.gov/brfss/annual_data/annual_2012.html) | Obesity rate |
|  | Diabetes rate |

*(*) - Variables newly added to the analysis*

| **3a. Adaptive capacity / Sensitivity - socioeconomic variables** | | | | | |
| --- | --- | --- | --- | --- | --- |
| Category | Sub-category | Source | Period | Resolution | Coverage |
| Housing | Occupants per room, Multi-unit housing,  Household type, Median year structure built,  Housing units by tenure, | [American Community Survey](https://www.census.gov/programs-surveys/acs) | 2005-2019 | As small as  census tract  / block-group level | U.S. |
| Income | Average income earned per person (Per capita income), Median household income,  Median gross rent, Median house value,  Households earning $10,000 or less,  Households earning $200,000 or more,  Households receiving food stamps/SNAP,  Tenure by household size |  |  |  |  |
| Occupation | Farming, fishing, mining, and forestry,  Construction and extraction,  Installation, maintenance, and repair,  Services |  |  |  |  |
| Employment | Unemployment rate, Labor force,  Below poverty level |  |  |  |  |
| Facilities | Telephone service available,  Computer and internet use, Kitchen |  |  |  |  |
| Transportation | Vehicles available |  |  |  |  |
| Language | Speak English “less than well” |  |  |  |  |
| Education | No high school diploma |  |  |  |  |
| Neighborhood | Healthy food accessibility | [Washington tracking network](https://fortress.wa.gov/doh/wtn/WTNPortal/home) | 2009 | Census tract | WA |
|  | Poor land use mix |  | 2010 |  |  |
|  | Children’s Park access |  | 2015 |  |  |
|  | Heavy traffic roadways |  | 2017 |  |  |
|  | Alcohol outlet density |  | 2009-2013 |  |  |
|  | Unoccupied housing |  | 2009-2017 |  |  |

**Table S3: Adaptive capacity and sensitivity metrics available in existing data sources relevant to the Western U.S.**

| **3b. Adaptive capacity - healthcare and community facilities** | | | | | |
| --- | --- | --- | --- | --- | --- |
| Category | Sub-category | Source | Period | Resolution | Coverage |
| Emergency preparedness and response | Emergency operations coordination,  Information sharing for emergency preparedness and response,  In-Home power dependent Equipment,  In-Home services requiring power,  Medical countermeasures dispensing,  Medical surge,  Non-Pharmaceutical interventions,  Public health surveillance and epidemiological investigation,  Public information and warning,  Medicare beneficiaries | [Washington tracking network](https://fortress.wa.gov/doh/wtn/WTNPortal/home) | 2016 | County | WA |
|  |  |  |  | ZIP code |  |
| Hospitals | [Total hospitals](https://www.kff.org/other/state-indicator/total-hospitals/),  [Hospitals by ownership type](https://www.kff.org/other/state-indicator/hospitals-by-ownership/),  [Total hospital beds](https://www.kff.org/other/state-indicator/total-hospital-beds/),  [Hospital beds per 1,000 population by ownership type](https://www.kff.org/other/state-indicator/beds-by-ownership/),  [ICU beds](https://www.kff.org/other/state-indicator/icu-beds/) | [Kaiser Family Foundation](https://www.kff.org/statedata/) | 2018 | State | U.S. |
| Hospital utilization | [Hospital admissions per 1,000 population by ownership type](https://www.kff.org/other/state-indicator/admissions-by-ownership/),  [Hospital emergency room visits per 1,000 Population by ownership type](https://www.kff.org/other/state-indicator/emergency-room-visits-by-ownership/),  [Hospital inpatient days per 1,000 population by ownership type](https://www.kff.org/other/state-indicator/inpatient-days-by-ownership/),  [Hospital outpatient visits per 1,000 population by ownership type](https://www.kff.org/other/state-indicator/outpatient-visits-by-ownership/) |  |  |  |  |
| Nursing facilities | [Total number of certified nursing facilities](https://www.kff.org/other/state-indicator/number-of-nursing-facilities/),  [Total number of residents in certified nursing facilities](https://www.kff.org/other/state-indicator/number-of-nursing-facility-residents/),  [Average number of certified nursing facility beds](https://www.kff.org/other/state-indicator/average-number-of-certified-nursing-facility-beds/),  [Average nurse hours per resident day in all certified nursing facilities](https://www.kff.org/other/state-indicator/average-nurse-hours-per-resident-day-in-all-certified-nursing-facilities-2003-2014/),  [Distribution of certified nursing facilities by ownership type](https://www.kff.org/other/state-indicator/nursing-facilities-by-ownership-type/),  [Distribution of certified nursing facilities by certification type](https://www.kff.org/other/state-indicator/nursing-facilities-by-certification-type/),  [Distribution of certified nursing facility Residents by primary payer source](https://www.kff.org/other/state-indicator/distribution-of-certified-nursing-facilities-by-primary-payer-source/),  [Distribution of certified nursing facilities by affiliation](https://www.kff.org/other/state-indicator/distribution-of-certified-nursing-facilities-by-affiliation/),  [Distribution of certified nursing facilities by hospital-based status](https://www.kff.org/other/state-indicator/distribution-of-certified-nursing-facilities-by-hospital-based-status/),  [Certified nursing facility occupancy rate](https://www.kff.org/other/state-indicator/nursing-facility-occupancy-rates/),  [Percent of certified nursing facilities with Resident groups and family groups](https://www.kff.org/other/state-indicator/percent-of-certified-nursing-facilities-with-resident-groups-and-family-groups-2003-2014/),  [Average number of deficiencies per certified nursing facility](https://www.kff.org/other/state-indicator/avg-of-nursing-facility-deficiencies/),  [Percent of certified nursing facilities Receiving a deficiency for actual harm or jeopardy](https://www.kff.org/other/state-indicator/of-facilities-w-serious-deficiencies/),  [Percent of certified nursing facilities with deficiencies](https://www.kff.org/other/state-indicator/nursing-facilites-with-zero-deficiencies/),  [Percent of certified nursing facilities with top ten deficiencies](https://www.kff.org/other/state-indicator/percent-of-certified-nursing-facilities-with-top-ten-deficiencies-2014/) |  | 2019 |  |  |
| Physicians | [Professionally active physicians](https://www.kff.org/other/state-indicator/total-active-physicians/),  [Professionally active physicians by gender](https://www.kff.org/other/state-indicator/physicians-by-gender/),  [Professionally active primary care physicians by field](https://www.kff.org/other/state-indicator/primary-care-physicians-by-field/),  [Professionally active specialist physicians by field](https://www.kff.org/other/state-indicator/physicians-by-specialty-area/) |  | 2020 |  |  |
| Nurse practitioners and physician assistants | [Total number of nurse practitioners](https://www.kff.org/other/state-indicator/total-number-of-nurse-practitioners/),  [Total number of nurse practitioners, by gender](https://www.kff.org/other/state-indicator/total-number-of-nurse-practitioners-by-gender/),  [Total number of physician assistants](https://www.kff.org/other/state-indicator/total-number-of-physician-assistants/),  [Total number of physician assistants, by gender](https://www.kff.org/other/state-indicator/total-physician-assistants/),  [Nurse practitioner scope of practice laws](https://www.kff.org/other/state-indicator/total-nurse-practitioners/),  [Physician assistant scope of practice laws](https://www.kff.org/other/state-indicator/physician-assistant-scope-of-practice-laws/) |  |  |  |  |
|  |  |  | 2015 |  |  |
| Access to care | [Percent of adults without an usual place of medical care](https://www.kff.org/other/state-indicator/percent-of-adults-without-a-usual-place-of-medical-care/),  [Percent of adults who had not seen or talked to a general doctor in the past 12 months](https://www.kff.org/other/state-indicator/percent-of-adults-who-had-not-seen-or-talked-to-a-general-doctor-in-the-past-12-months/),  [adults who report not seeing a doctor in the past 12 months because of cost by gender](https://www.kff.org/other/state-indicator/could-not-see-doctor-because-of-cost/),  [adults who report not seeing a doctor in the past 12 months because of cost by race/ethnicity](https://www.kff.org/other/state-indicator/percent-of-adults-reporting-not-seeing-a-doctor-in-the-past-12-months-because-of-cost-by-raceethnicity/),  [Adults who report not having a personal Doctor/health care provider by gender](https://www.kff.org/other/state-indicator/percent-of-adults-reporting-not-having-a-personal-doctor/),  [Adults who report not having a personal Doctor/health care provider by race/ethnicity](https://www.kff.org/other/state-indicator/percent-of-adults-reporting-not-having-a-personal-doctor-by-raceethnicity/),  [Percent of children with a medical home](https://www.kff.org/other/state-indicator/children-with-a-medical-home/),  [Adults reporting unmet need for mental health treatment in the past year](https://www.kff.org/other/state-indicator/adults-reporting-unmet-need-for-mental-health-treatment-in-the-past-year/) |  | 2014 |  |  |
|  |  |  | 2018 |  |  |
|  |  |  | 2017 |  |  |
|  |  |  | 2017-2018 |  |  |
| Rural health clinics | [Number of Medicare certified rural health clinics](https://www.kff.org/other/state-indicator/total-rural-health-clinics/) |  | 2018 |  |  |
| Community health centers | [Community health center delivery sites and patient visits](https://www.kff.org/other/state-indicator/community-health-center-sites-and-visits/),  [Community health center patients by payer source](https://www.kff.org/other/state-indicator/chc-patients-by-payer-source/),  [Community health center revenues by payer source](https://www.kff.org/other/state-indicator/community-health-center-revenues-by-payer-source/) |  |  |  |  |
| Health care employment | [Total health care employment](https://www.kff.org/other/state-indicator/total-health-care-employment/),  [Health care employment as a percent of total employment](https://www.kff.org/other/state-indicator/health-care-employment-as-total/) |  |  |  |  |
| Health professional shortage areas | [Primary care Health Professional Shortage Areas (HPSAs)](https://www.kff.org/other/state-indicator/primary-care-health-professional-shortage-areas-hpsas/),  [Mental Health Care Health Professional Shortage Areas (HPSAs)](https://www.kff.org/other/state-indicator/mental-health-care-health-professional-shortage-areas-hpsas/) |  | 2019 |  |  |

| **3c. Sensitivity - demographic variables** | | | | | |
| --- | --- | --- | --- | --- | --- |
| Category | Sub-category | Source | Period | Resolution | Coverage |
| Total population |  | [American Community Survey](https://www.census.gov/programs-surveys/acs) | 2005-2019 | As small as  census tract  / block-group level | U.S. |
| Age | Median age,  < 5 (17), ≥ 65 (85) |  |  |  |  |
| Sex | Female |  |  |  |  |
| Race/ethnicity | White, Black, Asian, other races, Hispanic, indigenous population, group quarters population |  |  |  |  |
| Disability |  |  |  |  |  |
| Nursing homes | ≥ 65 (85) in nursing facilities |  |  |  |  |
|  | Household type for children under 18 years in households |  |  |  |  |

| **3d. Sensitivity - underlying health conditions** | | | | |
| --- | --- | --- | --- | --- |
| Category | Source | Period | Resolution | Coverage |
| All causes of Death | [CDC wonder](https://wonder.cdc.gov/) | 1999-2018 | County | U.S. |
| Certain infectious and parasitic diseases |  |  |  |  |
| Neoplasms |  |  |  |  |
| Diseases of the blood and blood-forming organs and certain disorders involving the immune mechanism |  |  |  |  |
| Endocrine, nutritional, and metabolic diseases |  |  |  |  |
| Mental and behavioral disorders |  |  |  |  |
| Diseases of the nervous system |  |  |  |  |
| Diseases of the eye and adnexa |  |  |  |  |
| Diseases of the ear and mastoid process |  |  |  |  |
| Diseases of the circulatory system |  |  |  |  |
| Diseases of respiratory system |  |  |  |  |
| Diseases of digestive system |  |  |  |  |
| Diseases of the skin and subcutaneous tissue |  |  |  |  |
| Diseases of the musculoskeletal system and connective tissue |  |  |  |  |
| Diseases of the genitourinary system |  |  |  |  |
| Pregnancy, Childbirth, and the puerperium |  |  |  |  |
| Certain conditions originating in the perinatal period |  |  |  |  |
| Congenital malformations, deformations and chromosomal abnormalities |  |  |  |  |
| Symptoms, signs and abnormal clinical and laboratory findings, not elsewhere classified |  |  |  |  |
| Codes for special purposes |  |  |  |  |
| External causes of morbidity and mortality |  |  |  |  |
| Birth weight |  |  |  |  |
| BMI for Ages 20 + | [Washington tracking network](https://fortress.wa.gov/doh/wtn/WTNPortal/home) | 2014 | Census tract | WA |
| BMI for Ages 16 - 19 |  | 2014 |  |  |
| Cancer death |  | 2014-2018 |  |  |
| Cardiovascular disease mortality |  |  |  |  |
| Low birth weight |  |  |  |  |
| Premature death |  |  |  |  |
| Low life expectancy at birth |  |  |  |  |
| Reproductive and Birth Outcomes (low birthweight, very low birthweight, premature births, very premature births, infant mortality) | [Oregon environmental public health tracking](https://www.oregon.gov/oha) | 2000-2017 | County | OR |
| Heart Attack Hospitalizations (# of hospitalization, age-adjusted rate, crude rate) |  |  |  |  |
| Low Birth Weight | [CalEnviroScreen](https://oehha.ca.gov/calenviroscreen) | 2006-2012 | Census tract | CA |
| Cardiovascular Disease |  | 2011-2013 |  |  |

**Supplementary Text 2: Outdoor Working Populations**

Workers implementing forest management strategies are uniquely exposed to wildland fire smoke both during and after working hours. The number of workers implementing forest management practices, including firefighting or prescribed burns, is difficult to estimate because The U.S. Bureau of Labor Statistics Standard Occupational Classification System does not distinguish between municipal and wildland firefighters. A large number of forest fire personnel are in support roles at fire camps, mainly as contractors, and agency employers are large and cross-disciplined and the work is seasonal (*156*). An additional subgroup of populations fighting fires are incarcerated workers who have high physical demands with little or no control over their work.

The Bureau of Labor Statistics May 2019 report simply lists 420 ‘forest fire inspectors and prevention specialists’ in CA and OR (*157*). Nationally, the two largest employers of wildland firefighters are the US Forest Service and the US Department of the Interior, which includes the National Park Service, the Bureau of Land Management, and the Bureau of Indian Affairs. At the state level, the CA Department of Forestry and Fire Protection (8000 total employees), the CA Conservation Corps (3000 Corps Members), the Oregon Department of Forestry (no estimate), and the WA Department of Natural Resources (1350 permanent and temporary firefighters) are the largest employers for these western states. State Departments of Corrections account for 2200 and 300 fire-line personnel in CA and WA, respectively. Numerous counties, local entities, and private vendors employ wildland firefighters as well. A large number of forest fire personnel are on support roles in fire camps, mainly as contractors.

Nation-wide, farming, fishing, and forestry occupations, including wildland firefighters, are 47.6% Latinx, 1.8% Asian, and 4.4% African American (*157*). A subset of workers, as well as private landowners, in occupations including forest firefighter, forest technician, forest fire inspector, conservation technician, and supervisor engage in mechanical fuel management in addition to prescribed burning. The hand tools, mechanized equipment, and context of the work (increased exposure to noise and heat) in mechanical fuel management create occupational hazards such as those that can cause traumatic injuries and musculoskeletal disorders, all on top of the respiratory and cardiovascular hazards that come with smoke exposure (*158-161*).

**Supplementary Text 3: Policy Brief**

Understanding Policy Leverage Points for Integrating Public Health and Increased Use of Prescribed Burning in California, Oregon, and Washington

[Link to full report](https://www.nceas.ucsb.edu/sites/default/files/2021-02/Report_Understanding%20Policy%20Leverage%20Points%20for%20Integrating%20Public%20Health%20and%20Increased%20Use%20of%20Prescribed%20Burning%20in%20California%2C%20Oregon%2C%20and%20Washington.pdf)

| **People and Organizations** | |
| --- | --- |
| Organization | Source |
| Fire Adapted Communities Learning Network | https://www.nrs.fs.fed.us/pubs/jrnl/2010/nrs_2010_goldstein_001.pdf |
| Air Resource Advisors | https://sites.google.com/firenet.gov/wfaqrp-external/air-resource-advisors?authuser=0 |
| Prescribed Fire Councils | http://waprescribedfire.org/ |
| Collaborative Forest Landscape Restoration Program | https://www.fs.fed.us/restoration/documents/cflrp/CFLRP_LessonsLearnedCompiled20201016.pdf |
| The Lomakatsi Restoration Project | https://lomakatsi.org/ecological-approach-and-principles/ |
| California Fire MOU (Memorandum of Understanding) Partnership | https://www.sierraforestlegacy.org/CF_ManagingFire/FireMOU.php |
| State Health Departments | OR: https://www.oregon.gov/oha/ph/pages/index.aspx  WA: https://www.doh.wa.gov/  CA: https://www.cdph.ca.gov/ |
| Clean air agencies | OR: https://www.oregon.gov/deq/pages/air-n-water.aspx  WA: https://ecology.wa.gov/About-us/Our-role-in-the-community/Partnerships-committees/Clean-air-agencies  CA: ww2.arb.ca.gov |
| State Departments of Labor | OR: https://www.oregon.gov/BOLI/Pages/index.aspx  WA: https://lni.wa.gov/  CA: https://www.labor.ca.gov/ |
| State Environmental Departments | OR: https://www.oregon.gov/DEQ/Pages/index.aspx  WA: https://ecology.wa.gov/  CA: https://calepa.ca.gov/ |
| Tribal Entities | OR:https://www.oregon.gov/DHS/ABOUTDHS/TRIBES/Pages/Tribes.aspx  WA: https://www.washingtontribes.org/  CA: https://www.tasin.org/ |
| EPA | https://www.epa.gov/air-research/wildland-fire-research-protect-health-and-environment |
| Community Health Centers, Rural Health Care Networks | See Supplemental Text 3 |

**Table S4: List of available tools/resources for community preparedness & education.**

| **Tools and Resources** | |
| --- | --- |
| Tool or Resource | Source |
| Bluesky | https://portal.airfire.org/ |
| AirNow | https://fire.airnow.gov/ |
| AIRPACT | http://lar.wsu.edu/airpact/ |
| CalEnviroscreen | https://oehha.ca.gov/calenviroscreen/calenviroscreen-faqs |
| Washington Tracking Network | https://www.doh.wa.gov/DataandStatisticalReports/WashingtonTrackingNetworkWTN |
| Wildland Fire Decision Support System | https://wfdss.usgs.gov/wfdss/WFDSS_About.shtml |
| [WA State Environmental Health Disparities Map](https://www.doh.wa.gov/DataandStatisticalReports/WashingtonTrackingNetworkWTN/InformationbyLocation/WashingtonEnvironmentalHealthDisparitiesMap) | https://fortress.wa.gov/doh/wtn/WTNIBL/ |
| OR Vulnerability Assessment | https://www.oregon.gov/oha/ph/HealthyEnvironments/climatechange/Pages/Climate-Ethics-and-Health-Equity.aspx |
| EPA AWI | https://www.airnow.gov/aqi/aqi-basics/ |
| Washington Air Quality Advisory | https://ecology.wa.gov/Research-Data/Monitoring-assessment/Washington-Air-Quality-Advisory |
| British Columbia IQ Air | https://www.iqair.com/us/canada/british-columbia |
| County Health Rankins | https://www.countyhealthrankings.org/app/oregon/2020/rankings/jefferson/county/outcomes/overall/snapshot |
| Smoke Sense | https://www.epa.gov/air-research/smoke-sense-study-citizen-science-project-using-mobile-app |
| EPA EJ Screen | https://ejscreen.epa.gov/mapper/ |
| WRF Chem SFIRE | http://demo.openwfm.org/sj/ |

**Supplementary References**

(numbering continued from Main Text)

145. Abatzoglou, J. T. (2013). Development of gridded surface meteorological data for ecological applications and

modelling. *International Journal of Climatology*, *33*(1), 121-131.

146. Abatzoglou, J. T., Juang, C. S., Williams, A. P., Kolden, C. A., & LeRoy Westerling, A. (2020). Increasing

synchronous fire danger in forests of the western United States. *Geophysical Research Letters*, e2020GL091377.

147. Appel, K. W., Bash, J. O., Fahey, K. M., Foley, K. M., Gilliam, R. C., Hogrefe, C., Hutzell, W. T., Kang, D., Mathur,

R., Murphy, B. N., Napelenok, S. L., Nolte, C. G., Pleim, J. E., Pouliot, G. A., Pye, H. O. T., Ran, L., Roselle, S. J., Sarwar, G., Schwede, D. B., Sidi, F. I., Spero, T. L., and Wong, D.C.: The Community Multiscale Air Quality (CMAQ) Model Versions 5.3 and 5.3.1: System Updates and Evaluation, Geosci. Model Dev. Discuss. [preprint], https://doi.org/10.5194/gmd-2020-345, in review, 2020.

148. Appel KW, Napelenok S, Hogrefe C, Pouliot G, Foley KM, Roselle SJ, et al. 2018. Overview and evaluation of the

Community Multiscale Air Quality (CMAQ) Modeling System Version 5.2. In: Air Pollution Modeling and Its Application XXV. Springer International Publishing AG, Cham (ZG), Switzerland, 69–73,

<https://doi.org/10.1007/978-3-319-57645-9_11>.

149. Appel KW, Napelenok S, Foley KM, Pye HOT, Hogrefe C, Luecken D, Bash JO, Roselle SJ, Pleim JE, Foroutan H,

Hutzell W, Pouliot G, Sarwar G, Fahey K, Gantt B, Gilliam RC, Kang D, Mathur R, Schwede D, Spero T, Wong DC, Young J (2017) Overview and evaluation of the Community Multiscale Air Quality (CMAQ) model version 5.1.
Geoscience Model Development 10, 1703–1732. doi:10.5194/GMD-2016-226

150. Appel KW, Pouliot GA, Simon H, Sarwar G, Pye HOT, Napelenok SL, Akhtar F, Roselle SJ (2013) Evaluation of

dust and trace metal estimates from the Community Multiscale Air Quality (CMAQ) model version 5.0. Geoscientific Model Development 6, 883–899. doi:10.5194/GMD-6-883-2013

151. Byun D, Schere KL (2006) Review of the governing equations, computational algorithms, and other components of

the Models-3 Community Multiscale Air Quality (CMAQ) modeling system. Applied Mechanics Reviews 59, 51–77. doi:10.1115/1.2128636

152. Sullivan DC, Raffuse SM, Pryden DA, Craig KJ, Reid SB, Wheeler NJ, Strand T (2008) Development and

applications of systems for modeling emissions and smoke from fires: the BlueSky smoke modeling framework and SMARTFIRE. In ‘17th International Emissions Inventory Conference’, 2–5 June 2008, Portland, OR, USA. pp. 2–5. Available at https://www3.epa.gov/ttn/chief/conference/ei17/session12/raffuse_pres.pdf

153. Raffuse SM, Sullivan DC, Chinkin LR, Pryden DA, Wheeler NJ, Larkin NK,  Soja A (2007) Integration and

reconciliation of satellite-detected and Incident Command-reported wildfire information in the BlueSky Smoke Modeling Framework. In ‘Proceedings of the 6th Annual CMAS Conference’, 1–3 October 2007, Chapel Hill, NC, USA. (Community Modeling and Analysis System) Available at <https://www.cmascenter.org/conference/2007/abstracts/wheeler_session4_2007.pdf>

154. Raffuse SM, Larkin NK, Lahm PW, Du Y (2012) Development of version 2 of the wildland fire portion of the National

Emissions Inventory. Available at [www.epa.gov/ttn/chief/conference/ei20/session2/sraffuse.Pdf](http://www.epa.gov/ttn/chief/conference/ei20/session2/sraffuse.Pdf)

155. Ottmar RD, Sandberg DV, Riccardi CL, Prichard SJ (2007) An overview of the fuel characteristic classification

system – quantifying, classifying, and creating fuelbeds for resource planning. Canadian Journal of Forest Research 37, 2383–2393. doi:10.1139/X07-077

156. U.S. Bureau of Labor Statistics “Monthly Labor Review”, (Washington, DC, 2018)

157. U.S. Bureau of Labor Statistics “Monthly Labor Review”, (Washington, DC, 2019)

158. Britton C, Lynch CF, Ramirez M, Torner J, Buresh C, Peek-Asa C. Epidemiology of injuries to wildland firefighters.

Am J Emerg Med. 2013 Feb;31(2):339-45. doi: 10.1016/j.ajem.2012.08.032. Epub 2012 Nov 15. PMID: 23158597.

159. Semmens, E.O., Domitrovich, J., Conway, K. and Noonan, C.W. (2016), A cross‐sectional survey of occupational

history as a wildland firefighter and health. Am. J. Ind. Med., 59: 330-335. <https://doi.org/10.1002/ajim.22566>

160. S. A. Henn, C. Butler, J. Li, A. Sussell, C. Hale, G. Broyles, T. Reinhardt (2019) Carbon monoxide exposures

among U.S. wildland firefighters by work, fire, and environmental characteristics and conditions, Journal of Occupational and Environmental Hygiene, 16:12, 793-803, DOI: [10.1080/15459624.2019.1670833](https://doi.org/10.1080/15459624.2019.1670833)

161. West MR, Costello S, Sol JA, Domitrovich JW. Risk for heat-related illness among wildland firefighters: job tasks

and core body temperature change. Occup Environ Med. 2020 Jul;77(7):433-438. doi: 10.1136/oemed-2019-106186. Epub 2020 Jan 29. PMID: 31996475.
